# Development and Characterization of LDPE/EVA Films Incorporating Carvacrol Essential Oil with Antifungal Activity

**DOI:** 10.3390/foods14122069

**Published:** 2025-06-12

**Authors:** Konstantinos Safakas, Georgia C. Lainioti, Pinelopi Koutsodima, Panagiota Stathopoulou, Athanasios Ladavos

**Affiliations:** 1 Department of Food Science & Technology, University of Patras, GR-30100 Agrinio, Greece; 2 Department of Sustainable Agriculture, University of Patras, GR-30100 Agrinio, Greece; up1052623@ac.upatras.gr (P.K.); panstath@upatras.gr (P.S.)

**Keywords:** active packaging, carvacrol, mechanical properties, low-density polyethylene, poly(ethylene-co-vinyl acetate), antioxidant activity, antifungal properties

## Abstract

The development of antimicrobial and antioxidant packaging materials is critical for improving food safety and extending shelf life. This study aimed to design and characterize low-density polyethylene (LDPE) and poly(ethylene-co-vinyl acetate) (EVA) films incorporating organically modified montmorillonite (OMt) nanocarriers loaded with carvacrol (C) and thymol (T) essential oil components. The incorporation of carvacrol and thymol into OMt was conducted through an evaporation/adsorption method without the use of organic solvents. In the next step, LDPE, EVA and OMtC or OMtT were melt-compounded in order to obtain films. Characterization of the bioactive nanocarriers and films was performed through X-ray diffraction (XRD), tensile testing, oxygen permeability measurements (OTR) and antioxidant assays. Films LDPE/EVA/OMtC and LDPE/EVA/OMtT showed improved mechanical strength and antioxidant activity, with IC_50_ values between 0.32 and 0.52 mg/mL. Film with component weight ratio LDPE/EVA/OMtC equal to 80/10/10 also demonstrated enhanced barrier properties and significantly inhibited fungal growth on baguette bread for up to 60 days. These findings highlight the potential of these bioactive films to improve the microbial safety and shelf life of bakery products.

## 1. Introduction

Food spoilage due to microbial contamination, involving fungal growth, remains one of the principal causes of food waste worldwide. According to the FAO (Food and Agriculture Organization), around one-third of all food produced is wasted, with microbial spoilage contributing notably. Specifically, fungal infection affects products such as bread, dairy and fruits, frequently leading to obvious mold within 3–5 days under normal storage environment. Food packaging has a fundamental position concerning food products’ protection from external environmental effects. The main objective of food packaging is to maintain the cost-effectiveness of food and ensure food safety with the lowest environmental impact. Traditional packaging provides a barrier to moisture and oxygen but lacks active functions for microbial growth inhibition. The development of active packaging materials incorporating natural antimicrobial agents has gained increasing attention in recent years as an effective strategy to improve food safety, inhibit microbial growth and extend the shelf life of perishable products [1,2]. These compounds have demonstrated broad-spectrum antimicrobial and antifungal activity through the disruption of microbial cell membranes and interference with the metabolic processes. This approach is especially important in fresh and extended shelf-life foods, where controlling freshness and preventing impairing factors such as moisture, oxygen levels and microbial growth is essential [3]. By employing systems such as antimicrobial films, oxygen scavengers and moisture regulators, active packaging increases food stability, reduces waste and meets consumer demands for safe and long-lasting products [4].

To effectively deliver these active agents, suitable packaging materials are essential. Among the materials used for such applications, low-density polyethylene (LDPE) and poly(ethylene-co-vinyl acetate) (EVA) are commonly used in food packaging due to their complementary properties, including flexibility, durability and barrier properties [5,6]. LDPE is a thermoplastic polymer impermeable to moisture and resistant to light and oxygen, which retains food freshness [7]. EVA enhances the film’s elasticity and adhesion and facilitates the dispersion of active compounds within the polymer matrix. When blended, LDPE and EVA offer a versatile platform suitable for incorporating natural bioactive agents, such as carvacrol and thymol, known for their strong antimicrobial properties.

Notable examples of such natural agents are carvacrol and thymol, two naturally occurring compounds found in oregano (*Origanum vulgare*) and thyme (*Thymus vulgaris*), that exhibit strong antimicrobial and antifungal properties [8]. Carvacrol shows effects on a wide range of antifungal activity by destroying microbial cell membranes (it interacts with the lipid bilayer of fungal cells, increasing membrane permeability), modifying ionic fluxes, inhibiting ergosterol synthesis, inducing oxidative stress and blocking critical pathways of essential enzymes [9]. It is known to be quite effective against many strains of bacteria and fungi that are hazardous to humans or that cause important losses in the economy [8]. Carvacrol can also have synergistic effects when combined with other agents with conventional antifungal action, improving treatment effectiveness and reducing toxicity [10]. Embedding these compounds into polymer matrices such as LDPE/EVA can improve their stability and enable controlled release [11,12]. Carvacrol is considered a safe compound despite the limited amount of data on its metabolism in humans [8]. There are a number of EOs that are considered safe for direct use in food or food packaging. The Food and Drug Administration (FDA) has recognized GRAS (Generally Recognized as Safe) EOs from thyme, basil, cinnamon, oregano and cloves. GRAS is used for food and food-related products [13]. EOs belong to this category when used in accordance with well-known safety procedures. The European Food Safety Authority (EFSA) in the European Union (EU) is responsible for assessing food ingredients’ safety, containing essential oils. European regulations allow the use of certain bioactive compounds, like carvacrol, thymol, eugenol, limonene, cinnamaldehyde, citral, carvone, vanillin and linalool as flavoring agents [14].

The incorporation of bioactive materials into the LDPE/EVA blend seems to be an efficient method to develop antifungal packaging that actively prevents mold growth, maintains freshness, reduces reliance on synthetic preservatives and generally prolongs the shelf life of products. In terms of formulation and processing, it is still demanding to achieve a uniform distribution of bioactive materials within the polymer matrix while maintaining the required mechanical and physicochemical properties. The rising demand among consumers for natural products in food packaging has led to the replacement of synthetic additives with natural substances like essential oils, polyphenols and other plant extracts [15,16]. Among the various natural antimicrobial agents, essential oils (EOs) have been widely studied due to their strong bioactive properties, including antibacterial, antifungal and antioxidant activities [17,18]. However, the application of EOs in food packaging is often limited by their high volatility, hydrophobic nature and sensitivity to environmental conditions, requiring suitable polymeric carriers for effective incorporation and controlled release [19].

One specific area where this technology shows promise is in the preservation of bakery products. Bread and bakery products are among the most consumed foods in the world, and in recent years, the supply chain has grown significantly. The need to lengthen the shelf life of baked goods is a main concern for the bakery industry [20]. Because of moisture and microbial spoilage, baked goods are very fragile [21]. In fact, mold growth in bread causes substantial food waste and poses a significant financial problem, while it remains one of the principal worries of consumers. Guaranteeing food safety and preserving freshness depend on efficient packaging solutions. EO components have been incorporated into polymers and used for bread and bakery packaging, showing positive effects in the reduction of fungal growth on bread and other baked items [22,23,24]. Moreover, using natural bioactive agents like carvacrol or thymol reduces the need for synthetic preservatives, aligning with clean-label trends.

This work contributes to food safety and sustainability advancement by demonstrating the potential of natural bioactive nanocarriers—specifically OMtC and OMtT—in active packaging systems. The incorporation of compounds like carvacrol into LDPE/EVA matrices leads to effective antifungal properties of films that decrease mold growth in bakery products, which is a key issue behind food spoilage and waste. Using essential oil components as natural antimicrobial agents supports the move away from synthetic preservatives, in line with clean-label concepts and the growing consumer need for safer, more natural foods. Additionally, the extension of bread’s shelf life via functional packaging is directly related to the reduction of food waste, a fundamental objective in global sustainability agendas. This work links materials science with real-world food preservation challenges, providing a scalable approach for industry applications.

Based on our knowledge so far, there is limited research on active packaging films concerning LDPE/EVA blends with essential oil components [6,25,26,27], whereas there is no information about LDPE/EVA blends loaded with carvacrol as an antifungal agent. This study aims to develop and evaluate LDPE/EVA films with carvacrol and thymol, anticipating improved mechanical performance and antifungal efficacy through the incorporation of OMtC/OMtT to enable their application in active food packaging. In the first step, the incorporation of carvacrol (C) and thymol (T) essential oil components into the organically modified montmorillonite (OMt) was conducted through the evaporation/adsorption method, an eco-friendly approach developed in our laboratory. This method allows the direct incorporation of essential oil components into clays without the use of organic solvents, which have detrimental effects on the environment and human health. The bioactive nanocarriers were then blended with LDPE/EVA polymers through an extrusion method and formed into films in order to prepare and characterize LDPE/EVA/OMtC and LDPE/EVA/OMtT films. The characterization was conducted in terms of the structural and mechanical properties of the films. Moreover, the research assessed the films’ oxygen permeability and antioxidant activity. The ability of the LDPE/EVA/OMtC film to inhibit fungal growth on bread was also tested over a 60-day storage trial.

## 2. Materials and Methods

### 2.1. Materials

Carvacrol (5-isopropyl-2-methylphenol), 98%; thymol (2-isopropyl-5-methylphenol), ≥98.5%; and DPPH (2,2-diphenyl-1-picrylhydrazyl) and poly(ethylene-co-vinyl acetate) with vinyl acetate, 40 wt. %, were purchased from Sigma-Aldrich (Aldrich, Steinheim, Germany) and used without further modification. The organically modified montmorillonite Nanomer^®^ I.44P, a surface-modified montmorillonite clay with 40 wt.% dimethyl dialkyl (C14–C18) amine, was produced by Nanocor Inc. (Hoffman Estates, IL, USA), supplied by Sigma-Aldrich (St. Louis, MO, USA) and used as received, without any further treatment. The solvent ethanol absolute was purchased from Merck (Merck KGaA, Darmstadt, Germany). Low-density polyethylene (DOWTM LDPE 352E) was kindly provided by Achaika Plastics S.A. (Aigio, Greece).

### 2.2. Preparation of OMtC and OMtT Hybrids

The incorporation of the bioactive compound carvacrol or thymol into OMt was conducted through an evaporation/adsorption method developed in our lab [28], a green methodology for the direct incorporation of EO components onto clays without the use of organic solvents or high temperatures. In summary, 10 g of thymol or carvacrol was used with 10 g of montmorillonite, resulting in a nominal mass ratio of 1:1. To be more specific, the bioactive compound was placed on a glass plate at the bottom of a sealed, heated chamber, while the clay was positioned on a separate plate above it. The chamber was then closed and maintained at 120 °C for 96 h. The conditions of 120 °C for 96 h used in the adsorption/loading procedure were selected based on an optimization study carried out in our laboratory. This process allowed intra-layer and surface adsorption of the EO through vapor-phase interaction with the clay, without the use of organic solvents or direct mixing, thereby supporting the green chemistry approach.

### 2.3. Preparation of LDPE/EVA/OMtC and LDPE/EVA/OMtT Films

LDPE, EVA and OMtC or OMtT were melt-compounded using a Haake Mini Lab II twin co-rotating extruder (ThermoScientific, ANTISEL S.A., Athens, Greece) at 160 °C with a screw speed of 100 rpm for 10 min to ensure homogeneous mixing. The processing temperature was chosen based on the melting point of the LDPE used. The resulting masterbatch was then processed using a Manual Hydraulic Press with heated platens (Specac, ANTISEL S.A., Athens, Greece), where it was pressed between two Teflon sheets at 120 °C and 2 tons of pressure for 2 min. The film was rapidly quenched in an ice-water bath to finalize its formation.

### 2.4. Characterization Techniques

#### 2.4.1. XRD Analysis

The obtained films were characterized using XRD to estimate the incorporation of bioactive compounds into the interlayer space of the nanocarrier. The analysis was conducted on an advanced Brüker D8 diffractometer (Bruker, Analytical Instruments, S.A., Athens, Greece) with CuKα radiation (λ = 1.541874 Å). The diffractometer operated at a constant temperature of 20 °C, with a monochromatic beam maintained at 40 kV and 40 mA. The interlayer distance (d-spacing) of the nanocarrier sheets was determined from the 001 reflection. Scanning parameters were set as follows: 2θ range of 2–20°, increment of 0.03°, PSD of 0.764 and a slit width of 0.6 mm. The selected XRD parameters—specifically, the 2θ range of 2–30° with a 0.03° step size—were chosen to capture the basal reflections (001) of montmorillonite (Mt) and organically modified montmorillonite (OMt), which typically appear at low angles (around 4–8° 2θ). This region is critical for evaluating changes in the interlayer spacing due to the incorporation of essential oils such as carvacrol and thymol. The fine step size (0.03°) was employed to enhance resolution and allow for accurate determination of peak shifts and potential broadening, which are indicative of intercalation phenomena in the clay structure. Each sample was carefully placed on glass sample holders, ensuring that its surface was at the same level as the instrument’s reference plane.

#### 2.4.2. Mechanical Analysis

Tensile tests were performed on a Shimadzu AX-G universal testing machine with a 5 kN load cell (Shimadzu, Asteriadis S.A., Athens, Greece), following ASTM D638 standards [29]. For each film, five dog-bone type V specimens were tested at a deformation rate of 30 mm/min, according to ASTM D882 [30]. Young’s modulus, tensile strength and elongation at break were determined. Statistical analysis, including the calculation of mean values and standard deviation, was conducted on the results from the tested specimens.

#### 2.4.3. Oxygen Permeability Measurements of LDPE/EVA/OMtC and LDPE/EVA/OMtT Films

The oxygen permeability of the membranes was measured with an O.P.A. 8001 oxygen permeability analyzer (Systech Illinois Instruments Co., Johnsburg, IL, USA). The tests were conducted according to ASTM D3985 [31] (23 °C and RH = 0%). The oxygen permeability coefficient (PeO_2_) for each membrane was calculated according to the following equation:

PeO_2_ = OTR × ∆x (1)

where OTR represents the oxygen transmission rate through the film (cm^3^ O_2_ STP × cm^−2^ film area × s^−1^), and ∆x is the average film thickness (cm) measured with a digital micrometer at five different positions on each sample. The average value was used in the calculations. The use of this equation is appropriate for our film systems because the OTR values are typically normalized to unit thickness to compare materials with slightly different dimensions. Including thickness in the calculation allows for a more accurate assessment of the intrinsic barrier properties of the material, independent of sample variability. A lower permeability coefficient corresponds to a higher barrier capacity.

#### 2.4.4. DPPH Radical Scavenging Assay

The DPPH (2,2-diphenyl-1-picrylhydrazyl) free-radical scavenging method was used for the determination of the antioxidant activity of the samples. In this method, the bleaching rate of the stable free radical DPPH• at a characteristic wavelength in the presence of the sample is detected [32,33,34]. At an initial stage, the preparation of ethanolic solutions of the sample (1 mg/mL) and DPPH (1 mM) was conducted, and then appropriate volumes of ethanol, the ethanolic sample solution and 400 μL of the EtOH-DPPH solution were mixed to a final volume of 4 mL. Samples were kept at room temperature (RT) in the dark for 20 h, and the absorbance at 517 nm was then recorded. The control solution, consisting of ethanol and DPPH, was also measured at 517 nm. All measurements were conducted in triplicate, and the results are presented as the mean average.

The radical scavenging activity (%) was calculated using the following equation:

(2) $$Inhibition(\%)=\frac{A_{0}-A_{S}}{A_{0}}\times 100$$

where *A*_0_ is the absorbance of the DPPH solution without sample (control), and *A*_S_ is the absorbance in the presence of the film extract. The IC_50_ value, defined as the concentration of extract required to scavenge 50% of DPPH radicals, was determined by plotting % inhibition against the extract concentration and fitting the data to a nonlinear regression model. The concentration corresponding to 50% inhibition was extracted from the fitted curve. A lower IC_50_ value signifies a stronger antioxidant effect.

### 2.5. In Situ Antifungal Activity in Bread Spoilage

Performing in situ antifungal activity tests in bread spoilage experiments will allow the evaluation of the effectiveness of the active packaging (carvacrol-loaded films) under realistic storage conditions—mimicking how consumers would store and use bread. White bread, in this case a baguette, was purchased from a local bakery, and the selection of white bread was made since it is highly susceptible to spoilage, specifically mold growth. Additionally, white bread has elevated moisture content and a porous crumb structure, which makes it very vulnerable to moisture and humidity fluctuations. Furthermore, white bread lacks the natural antimicrobial properties found in whole grain or sourdough bread, making it a more challenging product to preserve. The above-mentioned makes the white baguette a suitable and realistic model for testing the packaging’s ability for freshness preservation. For each experimental setup, one slice of baguette was placed in an individual sterile clamshell container manufactured from plastic polyethylene terephthalate (PET) material (dimensions: 9.5 cm × 10 cm × 3.5 cm) and was tightly sealed with PET lids to limit air exchange while still allowing for natural fungal growth. Two active films (LDPE/EVA/OMtC) were used per test—one placed at the bottom and the other at the top of the bread. Control samples (bread slices in PET containers without active films) were included for comparison. The experiments were conducted in a controlled environment chamber over 60 days at room temperature (25 ± 2 °C) with a relative humidity of 65%. Each clamshell container was opened briefly (~30 s) every 3 days to simulate real-world conditions (such as repeated opening in home or retail settings) and to visually inspect the bread. Each experimental condition was tested in triplicate (n = 3) to ensure reproducibility and reliability of the results. The evaluation of fungal growth was based on visual inspection, observing the bread surfaces for signs of natural fungal contamination to simulate real-world conditions. In this study, the primary aim was to monitor overall fungal growth as an indicator of spoilage. As such, no specific mold species identification was performed. However, any visible fungal colonies were recorded to provide a comprehensive understanding of spoilage over time.

### 2.6. Microbial Load of Bread Samples

The evaluation of the effectiveness of the bioactive packaging film (LDPE/EVA/OMtC_80/10/10) in preventing microbial growth on bread during storage was performed using microbiological assays to estimate the microbial load on the bread slices. Visual observations for microbial (especially mold) growth were also carried out on the bread samples as mentioned in Section 2.5.

To prepare the stock solution for each sample, a small bread portion was aseptically weighed and suspended in phosphate-buffered saline (PBS). The mixture was then homogenized using a commercial blender for 1 min and subsequently agitated in a shaker incubator at 160 rpm for 40 min. Serial 10-fold dilutions were performed up to 10^−7^.

Following dilution, 0.1 mL of each sample was evenly spread onto Luria–Bertani (LB) agar, a nutrient-rich medium commonly used for bacterial cultivation. The plates were incubated at 30 °C for 48 h, after which discrete bacterial colonies were counted. Bacterial concentrations were expressed as colony-forming units per gram (CFUs/g).

For total fungal count (TFC), 0.1 mL of each dilution was inoculated onto potato dextrose agar (PDA) plates. These were incubated at 28 ± 2 °C for five days. After incubation, the number of fungal colonies was manually counted and multiplied by the dilution factor. TFC was reported as CFUs per gram of bread. All experiments were conducted in triplicate, and results are presented as mean values.

### 2.7. Statistical Analysis

The data obtained from oxygen permeability, antioxidant activity and mold enumeration were expressed as mean ± standard deviation (SD), and all analyses were performed in triplicate. All mechanical test results were expressed as mean ± standard deviation (SD) based on at least three independent measurements. To determine the statistical significance of differences among the film formulations, a one-way analysis of variance (ANOVA) was performed, followed by Tukey’s honest significant difference (HSD) post-hoc test at a 95% confidence level (*p* < 0.05). Groupings from the Tukey test are presented in the figures using letters above the error bars, where different letters indicate statistically significant differences between samples. Statistical analyses were conducted using OriginPro 2022.

## 3. Results

### 3.1. XRD Characterization of the Prepared Films

**Figure 1:**
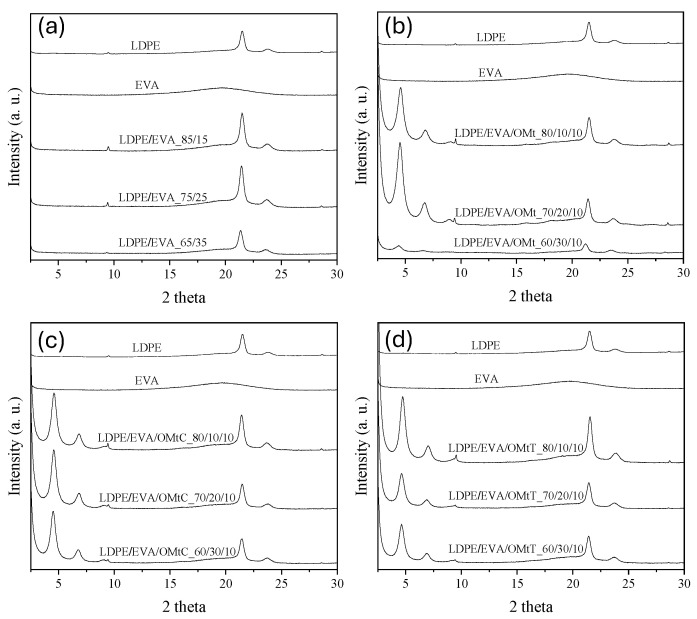
XRD patterns of films: (**a**) LDPE/EVA, (**b**) LDPE/EVA loaded with OMt, (**c**) LDPE/EVA loaded with OMt-carvacrol (OMtC) and (**d**) LDPE/EVA loaded with OMt-thymol (OMtT) bioactive nanocarriers.

X-ray diffraction (XRD) is an important tool for the analysis of the dispersion state of nanoclays in polymer matrices. It provides direct insight into the structure of the organoclay, to verify if the clay is intercalated, exfoliated, or agglomerated within the polymer matrix. Figure 1 shows the XRD patterns of all the prepared films, the LDPE/EVA mixtures (Figure 1a), LDPE/EVA/OMt (Figure 1b), as well as LDPE/EVA/OMtC (Figure 1c) and LDPE/EVA/OMtT films (Figure 1d). The interlayer spacing, *d*_001_ and the peak position (2θ) values for all the bioactive nanocarriers were calculated and presented in Table 1. As may be seen, LDPE shows two broad diffraction peaks around 2θ ≈ 21.4° (corresponding to the (110) plane) and 2θ ≈ 23.8° (corresponding to the (200) plane). EVA is less crystalline than LDPE and it tends to show even broader peaks or sometimes a halo pattern typical of amorphous materials. As shown in Figure 1, EVA shows one broad hump centered around 2θ ≈ 19–22°. XRD patterns for all nanocomposite films with or without bioactive compound are indicative of an intercalated nanocomposite structure where the multi-layer structure of the silicates is retained, with alternating polymer/silicate layers. The presence of 001 and, in most cases, 002 planes excludes the possibility of the formation of an exfoliated structure, where the primary particles of the organoclay are delaminated into individual nm-thick silicate layers.

As the EVA content increases, the LDPE/EVA/OMt films’ XRD pattern shows a small decrement at 2θ values of the 001 diffraction peak from 4.59° to 4.38° (Figure 1b), which corresponds to *d*_001_ of 19.3–20.2 Å. The same behavior is observed for the films that contain essential oils, LDPE/EVA/OMtT and LDPE/EVA/OMtC. These small variations in the interlayer distance of the clay may be due to the polarity of EVA, but, in any case, they are unable to affect the properties of the films.

The addition of OMt in the LDPE/EVA blends shows an increasing *d*_001_-spacing as the EVA content increases, indicating better dispersion of the additive within the polymer matrix. This trend suggests that EVA facilitates intercalation of polymer chains between clay layers due to its better compatibility with OMt. LDPE/EVA/OMtC films present slightly lower *d*_001_ values compared to LDPE/EVA/OMt, suggesting a more compact structure, probably due to stronger interactions between carvacrol and the clay layers. Moreover, LDPE/EVA/OMtC films exhibit higher *d*_001_ values compared to LDPE/EVA/OMtT, indicating greater interlayer spacing, probably due to better intercalation of the clay within the polymer matrix.

From the results of Table 1 and Figure 1, concerning the 2θ shifts and changes in *d*_001_-spacing, it is observed that the incorporation of bioactive additives alters the film’s internal structure. When the EVA content increases, this in general leads to increased interlayer spacing (*d*_001_), which can be attributed to improved intercalation of the clay layers. Variations in interlayer spacing consider different interactions between the polymer matrix and the bioactive compounds, leading to better dispersion and distribution of the bioactive agents, affecting the films’ physical and antimicrobial properties.

Moreover, comparing the results of the present work with those of Safakas et al. [24], it is evident that the addition of EVA to LDPE films with organically modified montmorillonite (PE/EVA/OMt) results in a shift of the 001 peak to higher 2θ angles compared to the corresponding films without EVA (PE/OMt), and the interlayer distance is reduced to less than 20 Å. A similar trend is observed in LDPE films with bioactive nanocarriers OMtC and OMtT after the addition of EVA. This suggests that EVA affects the dispersion and interaction of OMt within the polymer matrix. The observed reduction in d-spacing may be owed to the polar nature of EVA and its affinity for the surface of the organoclay. These polar-polar interactions possibly promote a more compact packing of the clay layers, leading to a more compact and stabilized nanostructure. This improved compatibility between EVA and OMt might contribute to advanced mechanical and barrier properties of the resulting composite films. Interestingly, this behavior contrasts with previous studies which reported an increase in interlayers with the addition of EVA to LDPE [35,36]. This discrepancy could be due to differences in OMt type, EVA composition (e.g., vinyl acetate content), or processing conditions, which may affect the final dispersion state of the nanoclay within the polymer.

### 3.2. Mechanical Properties of Films

Mechanical analysis is a characterization technique that provides valuable information about the strength, flexibility and durability of films. It helps to determine how the addition of EVA, OMt and carvacrol or thymol affects key parameters such as tensile strength, Young’s modulus and elongation at break. These insights are critical for material suitability assessment in order to be used in food packaging, where both mechanical integrity and flexibility are critical for performance and durability.

**Figure 2:**
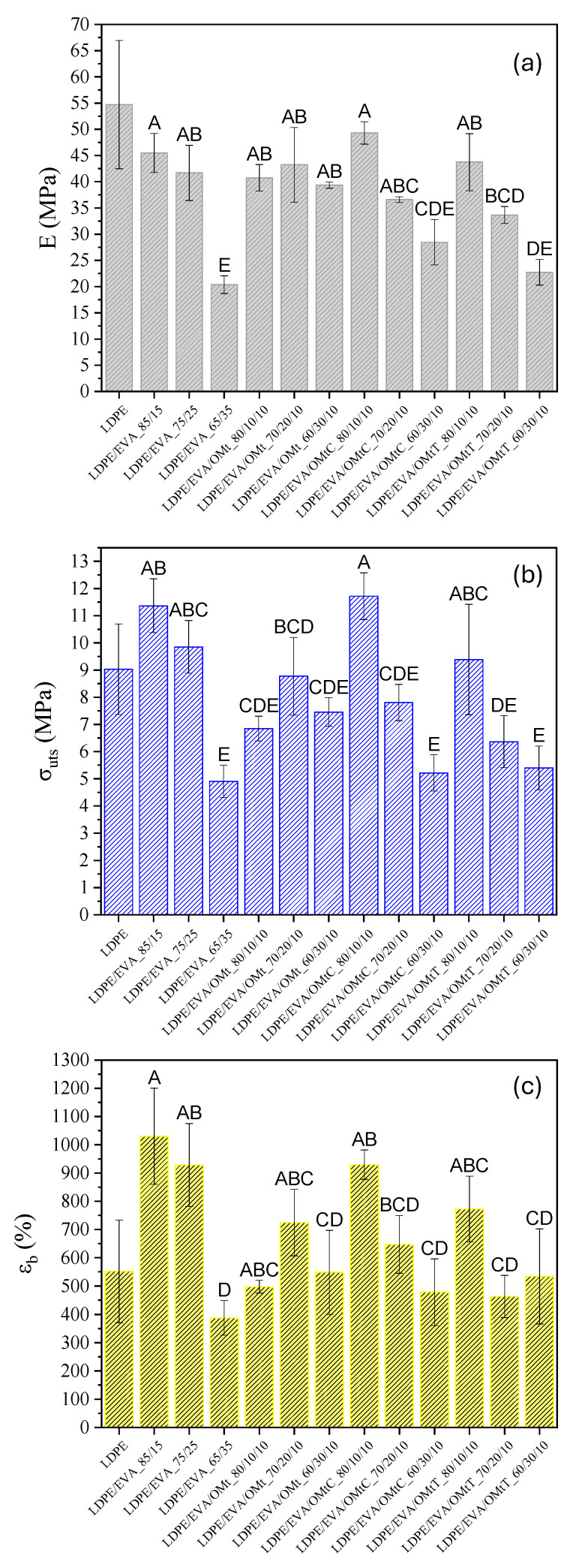
(**a**) Young’s modulus, (**b**) tensile strength (*σ*_uts_) and (**c**) strain at break (*ε*_b_) of LDPE/EVA films, LDPE/EVA films loaded with OMt and LDPE/EVA films loaded with OMt/carvacrol (OMt/C) and OMt/thymol (OMt/T) bioactive nanocarriers. Bars represent mean ± standard deviation. Different letters above the bars indicate statistically significant differences between samples (Tukey’s HSD test, *p* < 0.05).

Results of Young’s modulus (*E*), ultimate strength (*σ*_uts_) and elongation at break (*ε*_b_) obtained for all films are presented in Figure 2a–c, respectively. According to the results of Figure 2a, the increase of EVA content (from LDPE/EVA_85/15 to LDPE/EVA_65/35) leads to a decrease in Young’s modulus, which indicates that EVA acts as a plasticizer, making the films more flexible. The same behavior was found by Takidis et al. [37], where in the LDPE/EVA blends Young’s modulus decreases with an increasing amount of EVA-18. Since the crystallinity of EVA is lower than that of LDPE, it is also expected to have a lower elastic modulus. Crystalline regions can perform as physical crosslinks, leading to the enhancement of amorphous areas to restore storage stress. The incorporation of bioactive nanocarriers into the LDPE/EVA system did not significantly alter the overall decreasing trend. However, the sample LDPE/EVA/OMtC_80/10/10 showed the highest modulus value (49.30 MPa), which was statistically higher (group A) than most other formulations according to Tukey’s post-hoc test (*p* < 0.05). Films with 30 wt.% EVA showed the lowest modulus values, with LDPE/EVA/OMtC_60/30/10 and PE/EVA_65/35 being significantly lower (group D–E and E, respectively), reflecting a more than 50% reduction compared to pure LDPE. These differences were confirmed as statistically significant using one-way ANOVA followed by Tukey’s test, and different letter groupings are presented in the bar chart of Figure 2a to denote statistically distinct groups.

In Figure 2b, it appears that increasing EVA content leads to a decrease in ultimate tensile strength (*σ*_uts_), confirming that excessive EVA softens the film. This trend is statistically significant as verified by one-way ANOVA and Tukey’s post-hoc test (*p* < 0.05). According to the Tukey grouping, LDPE/EVA/OMtC_80/10/10 exhibited the highest *σ*_uts_ (11.72 MPa, group A), significantly higher than most other formulations (groups C–E), supporting its superior strength.

The LDPE/EVA/OMtC film at composition 80/10/10 also maintains a high *ε*_b_ value (929.75%), representing improved mechanical integrity, which can be attributed to strong polymer–clay interactions. In Figure 2c, it is observed that elongation at break initially increases, with the highest value recorded for LDPE/EVA_85/15 (*ε*_b_ = 1030.11%), followed closely by LDPE/EVA/OMtC_80/10/10 (*ε*_b_ = 929.75%) and LDPE/EVA_75/25 (*ε*_b_ = 928.29%). However, a further increase in EVA content (to 65/35) leads to a decrease in *ε*_b_ values, suggesting that excessive EVA may weaken the polymer network.

These trends are supported by statistical analysis using one-way ANOVA followed by Tukey’s post-hoc test (*p* < 0.05), with significant differences highlighted in the Tukey groupings shown in Figure 2c. LDPE/EVA_85/15 and LDPE/EVA/OMtC_80/10/10 fall into groups A and AB, indicating significantly higher elongation than samples like LDPE/EVA_65/35 (group D). The incorporation of OMt nanocarriers generally moderated *ε*_b_ values, with some formulations maintaining good ductility (e.g., LDPE/EVA/OMtT_80/10/10, group ABC), likely due to improved filler dispersion and interfacial compatibility.

The LDPE/EVA/OMtT films showed lower stiffness (*E*) and tensile strength compared to LDPE/EVA/OMtC films, indicating differences in the nanocarrier’s dispersion and interfacial interactions between the nanoclay and polymer matrix. OMtC probably promotes better intercalation due to its higher compatibility with the polymer blend, resulting in more effective stress transfer and enhanced mechanical reinforcement. In the case of OMtT-based systems, the lower performance could be the result of inadequate dispersion or weaker bond at the polymer–filler interface.

In other studies reported in the literature, the blend of EVA with LDPE matrices seemed to enhance flexibility and toughness due to the elastomeric nature of EVA. The study of Dadfar et al. [35] showed that the addition of EVA to LDPE improves the elongation at break and tensile strength of the films, making them more ductile and resilient. Moreover, the incorporation of organoclays, such as organically modified montmorillonite (OMt), into LDPE/EVA blends has been reported to further enhance mechanical properties. Zhong et al. [38] indicated that the addition of organoclay led to increased values of elastic modulus and tensile strength of the nanocomposite films. This was attributed to the reinforcement effect of well-dispersed clay platelets within the polymer matrix. Moreover, Pham et al. [39] investigated the mechanical properties of low-density polyethylene (LDPE) blended with ethylene-vinyl acetate (EVA) copolymer containing 28% vinyl acetate (EVA-28). They found that the addition of 10 wt.% EVA to LDPE increased elongation at break to 107.97% for the LDPE/10% EVA-28 blend, compared to 92.70% for pure LDPE, which corresponds to an approximate 16.5% increase.

However, the above-mentioned results are not absolute and may vary depending on factors such as the EVA content, the type and concentration of organoclay, as well as the processing conditions. Some studies have shown that high organoclay loadings may cause agglomeration, which can have an adverse effect on films’ mechanical properties. Thus, the optimization of the composition and the processing parameters is of utmost importance to achieve the required mechanical performance in LDPE/EVA blends loaded with organoclay nanocomposites.

### 3.3. Membrane Oxygen Permeability Measurements

The detrimental effects of oxygen on food quality are well documented in the literature [40,41]. Oxygen contributes to lipid oxidation, microbial growth, enzymatic browning and vitamin degradation. Therefore, the development of packaging materials that minimize or slow down food oxidation is essential for maintaining quality and extending shelf life. In the present work, the oxygen barrier performance of different LDPE/EVA-based films was tested in order to examine how the incorporation of EVA and bioactive nanocarriers (OMt, OMtC, OMtT) affects oxygen transmission rate (OTR) and permeability. The results are presented in Table 2.

According to the results presented in Table 2, pure LDPE had an oxygen permeability of 2.06 × 10^−8^ cm^2^/s. As EVA content increased (from LDPE/EVA_85/15 to 65/35), permeability also increased, with the highest value being 3.66 × 10^−8^ cm^2^/s at 65/35. This validates that EVA is more amorphous and less crystalline than LDPE, facilitating gas diffusion and reducing the barrier. The addition of OMt in the film LDPE/EVA/OMt_80/10/10 showed the lowest permeability (1.76 × 10^−8^ cm^2^/s), indicating improved barrier properties. As EVA content increased to 30%, a rise in permeability was observed (1.99 × 10^−8^ cm^2^/s), but the values were still lower in comparison to pure LDPE. This means that OMt nanosheets act as a gas diffusion barrier, decreasing permeability, although higher EVA content still weakens barrier properties. The observed decrease in permeability for films containing OMt indicates improved barrier properties due to better dispersion and possible intercalation of the clay platelets, forming a tortuous path for oxygen diffusion. These values are within the acceptable range for packaging semi-perishable products such as bakery and dairy goods. Moreover, the XRD results support the effective distribution and intercalation of the nanoclay particles within the polymer matrix. The placement of clay sheets, characterized by a high surface-to-volume ratio, between the polymer chains increases the tortuosity of the oxygen diffusion path. This structural arrangement significantly hinders gas permeability, effectively acting as an oxygen barrier. As a result, nanocomposite films demonstrated the highest oxygen-blocking efficiency among all the tested formulations.

When bioactive nanocarriers were added to LDPE/EVA blends, it seemed that permeability values increased in all cases. More specifically, LDPE/EVA/OMtC_80/10/10 had a permeability of 2.71 × 10^−8^ cm^2^/s, slightly worse than LDPE/EVA/OMt. With EVA content at 30% (LDPE/EVA/OMtC_60/30/10), permeability increased to 3.35 × 10^−8^ cm^2^/s. This behavior indicates that the bioactive nanocarrier OMtC influences the polymer matrix more than OMt and slightly decreases barrier properties.

When OMtT was added in LDPE/EVA blends, the permeability was 2.37 × 10^−8^ cm^2^/s for LDPE/EVA/OMtT_80/10/10, showing better behavior than OMtC-based films but worse than OMt-based films. At 30% EVA, permeability increased to 3.98 × 10^−8^ cm^2^/s, indicating a higher disruption of the polymer matrix than OMtC-based films, leading to the highest permeability values among all the films.

From the above results, it may be observed that the best barrier properties appeared in the LDPE/EVA/OMt_80/10/10 film, whereas the addition of bioactive nanocarriers weakened the films’ barrier performance, with the worst system being the LDPE/EVA/OMtT_60/30/10 film.

Farazi et al. [42] prepared LDPE/EVA blends with polyethylene graft copolymer with maleic anhydride (PE-G-MA), nanoclay (NC), potassium sorbate (KS) and garlic oil (GO) as antimicrobial substances. An increase in permeability values was observed for the systems LDPE/EVA/PE-MA+KS and LDPE/EVA/PE-MA+GO, and a decrease in permeability values for the system LDPE/EVA/PE-MA+GO+NC, compared to the LDPE/EVA/PE-MA system. Moreover, Dadfar et al. [35] showed that the addition of 6 wt% EVA to LDPE films led to a 6% increase in the permeability coefficient, and Marini et al. [43] showed that HDPE/EVA/nanoclay films with 15 wt% EVA and 5 wt% clay had an increased oxygen permeability coefficient of 20%.

In the literature, it has been shown that mixing LDPE with EVA can affect the oxygen permeability of the resulting films. Although EVA on its own may increase permeability due to its lower crystallinity, studies have shown that in nanocomposites, its integration may improve the dispersion of clay and increase interlayer spacing. This creates a more tortuous path for gas molecules, which reduces oxygen permeability and improves the barrier properties of the films [44].

The incorporation of OMt into LDPE/EVA blends has shown a further enhancement in barrier properties. The organoclay platelets have the ability to disperse into the polymer matrix, leading to increased tortuosity of the diffusion path for gas molecules. Better dispersion results in an effective reduction in the oxygen transmission rate, as gas molecules need to navigate a more complex pathway, thus improving the barrier performance of films [45].

### 3.4. Antioxidant Activity (AOA)

**Figure 3:**
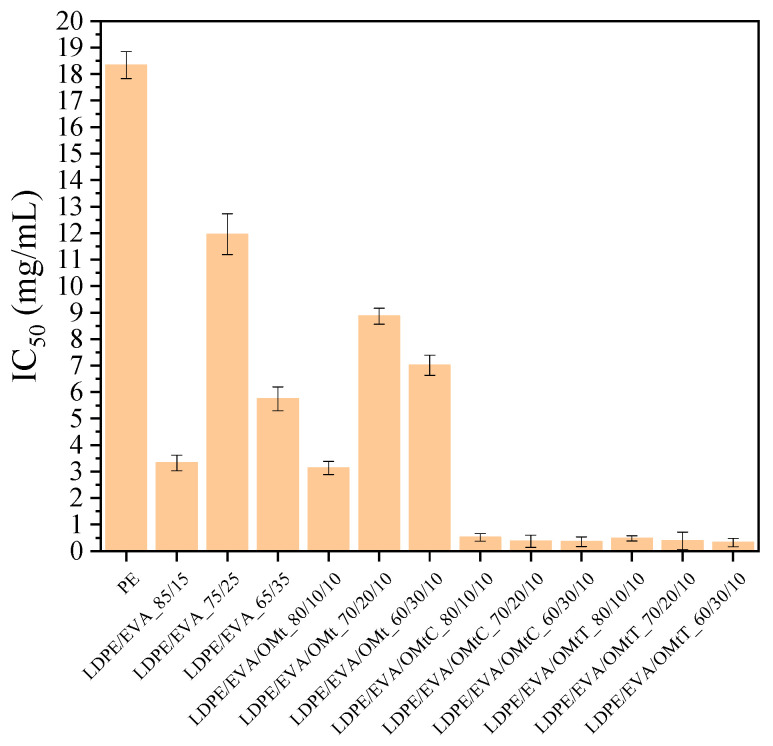
Antioxidant activity of LDPE films containing organically modified clay nanocarriers (OMt) infused with carvacrol (C) and thymol (T). Bars represent the mean ± standard deviation.

The role of antioxidant activity in food packaging is crucial for ensuring food safety. Research indicates that different factors, such as temperature, exposure to light, and the specific food simulant used, can influence the effectiveness of antioxidants in packaging materials [46]. In order to determine the ability of bioactive nanocomposite membranes to control oxidation in food, the antioxidant activity of the membranes was measured using the DPPH method [47]. The results are shown in Figure 3, where the IC_50_ values represent the concentration of the film required to inhibit 50% of free radicals; this means that lower values of IC_50_ indicate stronger antioxidant activity.

As shown in Figure 3, LDPE has the highest value of IC_50_ (18.33 mg/mL), indicating the weakest antioxidant activity. The incorporation of EVA in the mixtures leads to lower values of IC_50_, suggesting enhancement of the antioxidant activity of the films. More specifically, LDPE/EVA films with the compositions 85/15 and 65/35 show improved antioxidant activity, with IC_50_ values of 3.33 mg/mL and 5.75 mg/mL, respectively, whereas the 75/25 composition shows fluctuating behavior. This suggests that EVA contributes positively to the radical scavenging ability of the blend.

The LDPE/EVA/OMt films exhibit IC_50_ values between 3.14 and 8.86 mg/mL, showing moderate antioxidant activity and demonstrating that the incorporation of OMt contributes to free radical inhibition. Finally, films with the bioactive nanocarriers LDPE/EVA/OMtC and LDPE/EVA/OMtT showed the lowest values of IC_50_. Specifically, the IC_50_ values of 0.32–0.52 mg/mL observed for these films, which are significantly lower than those of the LDPE (18.33 mg/mL) and LDPE/EVA control films, support their superior antioxidant performance. This improvement is attributed to the presence of carvacrol and thymol, two phenolic compounds known for their effective radical scavenging due to their hydroxyl functional groups. The observed results highlight the synergistic effect of EVA and essential oil-loaded clays in significantly enhancing the antioxidant properties of LDPE-based films, making them highly suitable for active food packaging applications.

Table 3 presents the variation in antioxidant activity of LDPE/EVA/OMtC and LDPE/EVA/OMtT films, with the compositions 80/10/10, 70/20/10 and 60/30/10, relative to pure LDPE after one month of storage in a sealed container and in an open environment. The results of Table 3 suggest that all LDPE/EVA/OMtC and LDPE/EVA/OMtT films exhibited a +97% to +99% increase relative to the performance of LDPE, verifying the effectiveness of OMtC and OMtT bioactive nanocarriers. Moreover, all the films seem to maintain their enhanced properties when stored in a sealed bag or under environmental conditions. LDPE/EVA/OMtC films with 60/30/10 composition showed the highest percentage change (+99%) when stored in an open environment, possibly demonstrating slightly better retention of properties under these conditions.

### 3.5. Food Preservation and Fungal Growth Assessment

The prepared LDPE/EVA/OMtC_80/10/10 film was applied for baguette preservation in order to test its potential for food conservation. The film was applied by means of a standard method of surface treatment of the bread (coating), and in this way, it does not require special implementations by the packaging transforming industry, and it can be applied without modifying its processes.

**Figure 4:**
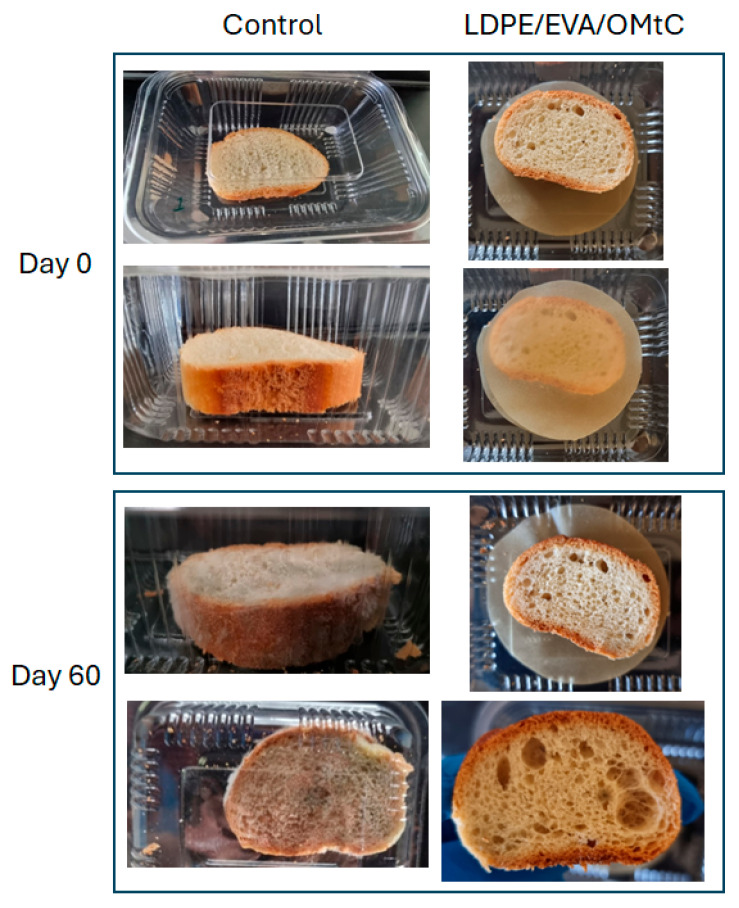
Assessment of mold growth in white baguette bread covered with LDPE/EVA/OMtC_80/10/10 film or with no film (control).

For testing purposes, one slice of baguette was placed in a commercial plastic package (polyethylene), with or without (control) the prepared LDPE/EVA/OMtC_80/10/10 film, and sealed for 60 days at 25 °C. The samples were visually observed for 60 days, and the fungal growth intensity is shown in Table 4. Images in Figure 4 illustrate the white baguette at the beginning of the experiment (Day 0) and after 2 months (Day 60) of storage.

**Figure 5:**
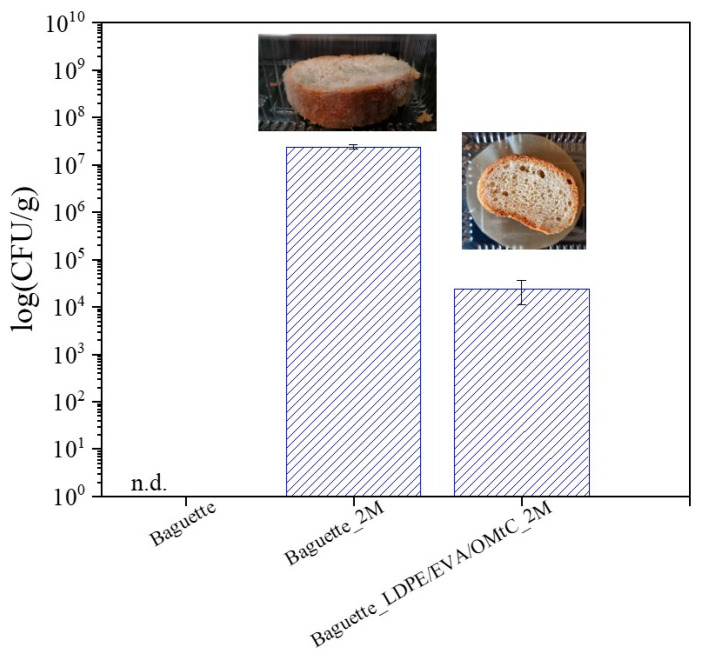
Mold count on baguette during storage for 0 days and 60 days (2M) at 25 °C and 65% RH without film and with the active film LDPE/EVA/OMtC_80/10/10. Bars represent the mean ± standard deviation. n.d.: not determined.

Microbial spoilage, and predominantly fungal contamination, remains the most important contributor to substantial waste in packaged bakery products. In this line, microbiological analysis was conducted on white baguette with LDPE/EVA/OMtC_80/10/10 film with or no film during storage at room temperature (25 ± 2 °C) for 60 days. No bacterial colonies were detected on the LB plates throughout the experiment; therefore, the focus shifted primarily to monitoring mold growth. The results showed no fungal coverage of the surface for the treated baguette with LDPE/EVA/OMtC_80/10/10 film and almost 50% fungal coverage on the control bread surface. The enumeration of fungal load results (CFU/bread g) is illustrated in Figure 5.

In our study, fungal counts were undetectable in freshly baked bread. Usually, ≤10^2^–10^3^ CFU/g (100–1000 CFU/g) is considered acceptable in fresh bread at the time of production or during early shelf life. In the treated baguette, and after a 60-day storage, the CFUs/g remained <0.5 × 10^4^, indicating spoilage beginning and post-production contamination. This low fungal load is highly relevant for both shelf-life extension and food safety. Shelf-life studies consider visible mold growth and microbial counts >10^3^–10^4^ CFU/g as spoilage thresholds. In bakery products such as white bread, visible mold growth typically becomes apparent once fungal populations exceed approximately 10^4^ to 10^5^ CFU/g [48]. In general, the shelf life of bread is 5–7 days at room temperature, 1–2 weeks in the refrigerator and 3 months in the freezer [49]. In our study, the bread without the tested coating revealed more than 10^7^ CFUs/g and was considered unsatisfactory or unacceptable for consumption. Bread wrapped in active membranes (LDPE/EVA/OMtC_80/10/10) demonstrated high effectiveness in delaying fungal growth, with fungal counts remaining within marginally acceptable microbial limits (10^4^ CFU/g) even after 60 days of storage (Figure 5). By keeping fungal contamination below this threshold, active packaging effectively delays the onset of visible spoilage, thereby prolonging the sensory and textural quality of the bread. This translates to a longer marketable shelf life, which is critical for reducing food waste and improving economic value for producers and retailers. In addition to the noticeable reduction in mold growth, the bread also maintained a fresher appearance over this long storage period. Moreover, fungal growth on bread can lead to the production of mycotoxins—secondary metabolites produced by certain mold species that pose serious health risks to consumers, including immunosuppression and carcinogenic effects [50]. Although our study did not identify specific mold species or directly measure mycotoxins, the substantial reduction in fungal counts suggests a lower risk of mycotoxin contamination, contributing to improved food safety. Additionally, maintaining fungal populations at such low levels supports product safety by reducing the risk of allergic reactions and respiratory issues associated with mold spores. The results presented in this study demonstrated that carvacrol-containing packaging (LDPE/EVA/OMtC_80/10/10) could be an effective natural alternative not only in preserving the quality and freshness of bread but also in enhancing its safety by mitigating spoilage and potential mycotoxin risks.

In freshly baked, unsealed bread such as the tested baguette, fungal growth typically begins on the surface—particularly in areas exposed to air. Mold tends to favor the soft, moist crumb and the outer crust, which retain higher humidity. Common fungal species observed include *Penicillium*, *Aspergillus* and *Rhizopus*. In particular, *Penicillium* spp. and *Aspergillus* spp. are able to grow on the bread surface and form a greenish-blue layer, similar to our observations. Fungal colonization typically presents as irregularly distributed patches on the crust or other exposed surfaces of the bread. In sliced bread, the interior crumb becomes exposed during cutting, increasing its vulnerability to airborne mold spores. This exposure accelerates fungal colonization, which often starts at the edges and progresses inward. In such cases, commonly observed fungi include *Penicillium*, *Aspergillus*, *Rhizopus* and *Mucor*, with the latter often presenting as a fluffy white mold.

It is worth noting that the most common technique for the prevention or control of fungal growth in food is the use of antifungal agents containing chemical composites that prevent or delay the development of fungi. Natural antimicrobials derived from plants [51] are the most important solution against chemical antimicrobials. EOs and their components extracted from plants or herbs can represent one of the most promising natural products against the growth of microorganisms [52,53,54].

EOs provide a natural substitute for bread preservation by suppressing or stopping the growth of dangerous fungi and mycotoxins in food [52]. Their chemical structure contains a variety of organic molecules, such as hydrocarbons, alcohols, aldehydes, esters, phenols, ketones and others. The mechanism of action of EOs, as well as the morphological alterations, is still unclear and can differ depending on the type of fungus and the components of the EO. Furthermore, the matrix in which they are used and the application technique also have an impact on their antimicrobial efficacy [21]. Antifungal activity depends on a number of factors, including the presence and location of hydroxyl groups, the existence of an aromatic ring, their fat solubility and their spatial arrangement.

The main mechanism of EOs’ antimicrobial or antifungal action may be attributed to the properties of terpenes/terpenoids, which can disrupt the cell membrane, due to their highly lipophilic nature and low molecular weight, leading to cell death or the prevention of sporulation and germination in spoilage molds [55].

The dispersion of OMt affects the stiffness and strength of the LDPE/EVA films by altering polymer–filler interactions. This structural impact also significantly controls the release of bioactive compounds like carvacrol, which control the antioxidant and antifungal effects. For example, LDPE/EVA/OMtC films displayed improved mechanical integrity and superior bioactivity, demonstrated by the lowest IC_50_ values (0.32–0.52 mg/mL), as well as a significant reduction in fungal growth in shelf-life studies. These findings suggest that optimal nanocarrier dispersion reinforces the matrix and provides a more uniform and sustained release of active compounds, improving radical scavenging activity and microbial inhibition.

From the antioxidant and antifungal results of the present study, we may conclude that there is strong accordance between them, showing that the active membranes are effective not only against oxidative degradation but also against fungal growth prevention. More specifically, the prolonged antioxidant activity of LDPE/EVA/OMtC and LDPE/EVA/OMtT films, with lower values of IC_50_ compared to neat LDPE/EVA and LDPE/EVA/OMt, suggests long-term stability and effectiveness in free radical scavenging. This behavior corresponds with the antifungal performance of membranes, where the LDPE/EVA/OMtC_80/10/10 film restrained fungal growth on baguettes, with microbial counts remaining within standard limits even after 60 days of storage. This is a very important outcome, showing that the active compounds in the membranes enhance both oxidative protection and microbiological resistance, which emphasizes their feasibility as multifunctional food packaging materials for long-term storage.

This dual functionality is very important for the use of these active membranes in food packaging as a function of time. It is known that essential oils have gained commercial interest in recent years in the production of bread and dough products due to their natural antifungal properties. In the study by Sharma et al. [56], poly (3-hydroxybutyrate-co-4-hydroxybutyrate) or P(3HB-co-4HB) incorporating thyme EO as active packaging was used for the extension of white bread’s shelf life, showing a 5-day extension compared to an extension of 1–4 days for the neat film. Ju et al. [57] showed that the use of a eugenol and citral EO mixture (1:1) in corn starch microcapsule sachets led to molds and yeasts decreasing from 100% to 56% at 25 °C and from 90% to 26% at 35 °C under storage conditions. Moreover, sliced bread stored in LDPE, PP and HDPE bags with the same essential oil sachets showed no mold development up to days 16, 14 and 14, respectively.

However, there are limited studies that have used EVA in combination with EO to test antifungal or antibacterial properties. Nostro et al. [25] studied the antibacterial and antibiofilm properties of EVA films with different concentrations (3.5 wt.% and 7 wt.%) of carvacrol or cinnamaldehyde essential oil constituents. They found that films with 7 wt.% EO showed a substantial bactericidal effect (reduction of 4 and 2 log CFU) against *Staphylococcus aureus* and *Escherichia coli* and a reduction of about 1 log CFU against *Staphylococcus epidermidis* and *Listeria monocytogenes*, whereas the biofilm formation was significantly lower than that developed at the control. Phala et al. [27] investigated the antifungal activity of 20 and 30 wt.% of spearmint essential oil or carvone, incorporated into EVA and linear LDPE strands, against citrus postharvest pathogens affecting kumquats, showing the preservation of kumquats over a 21-day period.

Our results demonstrate that packaging materials that balance structural performance and bioactive functionality can be created by carefully adjusting the type and content of nanocarriers. Because mechanical robustness, oxidation prevention and extended microbial shelf life are crucial requirements for packaging quality in real-world applications, this multifunctionality is especially pertinent. Similar findings were reported by Saadati Ardestani et al. [58], who incorporated carvacrol and thymol into PLA films via supercritical CO_2_-assisted impregnation and demonstrated strong antibacterial activity. Likewise, Esfandiari et al. [59] used supercritical fluid extraction and impregnation to incorporate rosemary essential oil into LLDPE films, resulting in significant antioxidant activity and structural stability, as evidenced by DPPH assays and IC_50_ values comparable to the free extract. While both studies used supercritical processing, our work introduced a solvent-free alternative using OMt-based nanocarriers to incorporate bioactive agents into LDPE/EVA blends through melt compounding. This method offers the additional advantage of clay reinforcement, which enhances not only antimicrobial efficacy but also mechanical properties. These complementary findings highlight the potential of diverse incorporation strategies—whether based on supercritical CO_2_ or nanoclay-assisted dispersion—for designing advanced active packaging materials.

To assess potential sensory impacts, a preliminary evaluation was conducted by three trained researchers. Aroma and taste intensity were compared across three samples: control baguette (unpackaged), baguette packaged with active LDPE/EVA/OMtC_80/10/10 film and the active LDPE/EVA/OMtC_80/10/10 film itself. On a 1–5 scale, the aroma scores were 1 for control baguette, 5 for the film alone and 1.5 for the packaged baguette—indicating minimal aroma transfer. In terms of taste, two researchers detected no difference in the packaged baguette, while one noted a slight oregano flavor, suggesting limited sensory influence from the bioactive film.

## 4. Conclusions

This study presents a comprehensive evaluation of LDPE/EVA-based bioactive nanocomposite films incorporating organically modified montmorillonite (OMt) carriers loaded with natural compounds such as carvacrol and thymol, aimed at producing multifunctional packaging for active food preservation. Our results confirm that, beyond enhancing mechanical strength, the films display antioxidant and antifungal properties, mainly extending bread shelf life up to 60 days.

The incorporation of antifungal agents directly into food packaging materials will contribute to the reduction in the requirement for synthetic preservatives, aligning with recent developments in sustainable food preservation. The results of the present work will support further studies and the use of bioactive films as a promising approach in order to improve food safety, reduce waste and comply with environmental standards in the food packaging industry.

These films present a promising alternative to conventional packaging by reducing the need for synthetic preservatives and extending shelf life. Their ability to reduce fungal growth and limit the necessity for synthetic preservatives supports current industry attempts toward cleaner-label packaging solutions.

Future research will focus on assessing the films’ performance with other food products, evaluating consumer acceptance and addressing regulatory concerns to enable their practical application in commercial food packaging.

## Figures and Tables

**Table 1:** XRD results of LDPE/EVA films.

Code	Film Composition (%)	2θ (°)	*d*_001_ (Å)
LDPE	100	-	-
EVA	100	-	-
LDPE/EVA/OMt	80/10/10	4.59	19.3
LDPE/EVA/OMt	70/20/10	4.53	19.5
LDPE/EVA/OMt	60/30/10	4.38	20.2
LDPE/EVA/OMtC	80/10/10	4.58	19.3
LDPE/EVA/OMtC	70/20/10	4.56	19.4
LDPE/EVA/OMtC	60/30/10	4.53	19.5
LDPE/EVA/OMtT	80/10/10	4.73	18.7
LDPE/EVA/OMtT	70/20/10	4.61	19.2
LDPE/EVA/OMtT	60/30/10	4.58	19.3

**Table 2:** Oxygen transmission rate (OTR), oxygen permeability coefficient (PeO_2_) and PEO/EVA membrane thickness values.

Code	Thickness (mm)	OTR (cc/m^2^/Day)	Permeability O_2_ (×10^−8^ cm^2^/s)
LDPE	0.1404	1268.18	2.06 ± 0.12
LDPE/EVA_85/15	0.1500	1570.00	2.68 ± 0.13
LDPE/EVA_75/25	0.1362	1709.00	2.68 ± 0.32
LDPE/EVA_65/35	0.1283	2467.30	3.66 ± 0.22
LDPE/EVA/OMt_80/10/10	0.1545	997.50	1.76 ± 0.13
LDPE/EVA/OMt_70/20/10	0.1616	1054.40	1.96 ± 0.23
LDPE/EVA/OMt_60/30/10	0.1437	1200.75	1.99 ± 0.01
LDPE/EVA/OMtC_80/10/10	0.1645	1421.25	2.71 ± 0.01
LDPE/EVA/OMtC_70/20/10	0.1353	2007.15	3.14 ± 0.07
LDPE/EVA/OMtC_60/30/10	0.1379	2100.30	3.35 ± 0.22
LDPE/EVA/OMtT_80/10/10	0.1399	1471.38	2.37 ± 0.02
LDPE/EVA/OMtT_70/20/10	0.1375	1830.75	2.91 ± 0.19
LDPE/EVA/OMtT_60/30/10	0.1345	2557.50	3.98 ± 0.07

**Table 3:** Variation in antioxidant activity of films stored for one month in a sealed bag or in an open environment (env), relative to LDPE.

Code	Increase (%)
LDPE/EVA/OMtC_80/10/10_1 Month	+97
LDPE/EVA/OMtC_80/10/10_1 Month env	+98
LDPE/EVA/OMtC_70/20/10_1 Month	+98
LDPE/EVA/OMtC_70/20/10_1 Month env	+98
LDPE/EVA/OMtC_60/30/10_1 Month	+98
LDPE/EVA/OMtC_60/30/10_1 Month env	+99
LDPE/EVA/OMtT_80/10/10_1 Month	+97
LDPE/EVA/OMtT_80/10/10_1 Month env	+97
LDPE/EVA/OMtT_70/20/10_1 Month	+98
LDPE/EVA/OMtT_70/20/10_1 Month env	+98
LDPE/EVA/OMtT_60/30/10_1 Month	+98
LDPE/EVA/OMtT_60/30/10_1 Month env	+98

**Table 4:** Assessment of visual observation of baguette stored for 60 days at 25 °C.

Bread Sample	Fungal Growth Intensity	
	Day 0	Day 60
Baguette	−	++
Baguette_PE/EVA/OMtC_80/10/10	−	−

Note: − absence of fungal growth, + fungal growth 25% of surface, ++ fungal growth 25–50% of surface.

## Data Availability

The original contributions presented in this study are included in the article. Further inquiries can be directed to the corresponding authors.
